# Divergence history and hydrothermal vent adaptation of decapod crustaceans: A mitogenomic perspective

**DOI:** 10.1371/journal.pone.0224373

**Published:** 2019-10-29

**Authors:** Shao’e Sun, Zhongli Sha, Yanrong Wang

**Affiliations:** 1 Deep Sea Research Center, Institute of Oceanology, Chinese Academy of Science, Qingdao, China; 2 Center for Ocean Mega-Science, Chinese Academy of Sciences, Qingdao, China; 3 Laboratory for Marine Biology and Biotechnology, Qingdao National Laboratory for Marine Science and Technology, Qingdao, China; 4 University of Chinese Academy of Sciences, Beijing, China; The Chinese University of Hong Kong, CHINA

## Abstract

Decapod crustaceans, such as alvinocaridid shrimps, bythograeid crabs and galatheid squat lobsters are important fauna in the hydrothermal vents and have well adapted to hydrothermal vent environments. In this study, eighteen mitochondrial genomes (mitogenomes) of hydrothermal vent decapods were used to explore the evolutionary history and their adaptation to the hydrothermal vent habitats. BI and ML algorithms produced consistent phylogeny for Decapoda. The phylogenetic relationship revealed more evolved positions for all the hydrothermal vent groups, indicating they migrated from non-vent environments, instead of the remnants of ancient hydrothermal vent species, which support the extinction/repopulation hypothesis. The divergence time estimation on the Alvinocarididae, Bythograeidae and Galatheoidea nodes are located at 75.20, 56.44 and 47.41–50.43 Ma, respectively, which refers to the Late Cretaceous origin of alvinocaridid shrimps and the Early Tertiary origin of bythograeid crabs and galatheid squat lobsters. These origin stories are thought to associate with the global deep-water anoxic/dysoxic events. Total eleven positively selected sites were detected in the mitochondrial OXPHOS genes of three lineages of hydrothermal vent decapods, suggesting a link between hydrothermal vent adaption and OXPHOS molecular biology in decapods. This study adds to the understanding of the link between mitogenome evolution and ecological adaptation to hydrothermal vent habitats in decapods.

## Introduction

Deep-sea hydrothermal vents are chemosynthetic ecosystems, which are characteristed by high temperature (up to 390°C), low oxygen levels, enriched hydrogen sulfide (H_2_S), methane (CH_4_) and heavy metals, such as iron, zinc, and copper [1]. Owning to their unusual chemistry, hydrothermal vents have been considered as the home to unique life forms [2]. In addition to chemoautotrophic bacteria, more than 600 animal species have been discovered in this extreme environment [3]. Decapod crustaceans, such as alvinocaridid shrimps, bythograeid crabs and galatheid squat lobsters, are important fauna in the hydrothermal vents, representing approximately 10% of all taxa reported from these vents [1, 4–5]. The discovery of these hydrothermal-vent faunas stimulates an increasing research effort to explore where the vent communities originated and when did they invade the hydrothermal vent habitat.

Two major hypotheses, the antiquity [6] and the extinction/repopulation hypothesis [7], has been proposed to explain the origin and distribution of vent fauna. Although most studies are consistent with the extinction/repopulation hypothesis [5, 8–9], the divergence time of the hydrothermal vent fauna always controversial. Shank et al (1999) revealed the Miocene origin (~10 Ma) of hydrothermal vent alvinocarid shrimp based on the evolutionary rate of the *cox1* gene [8]. Yang et al (2013) indicated the early Tertiary origin (48.4–55.9 Ma) of bythograeid crabs and the late Cretaceous/early Tertiary divergence (51.5–69.7 Ma) of alvinocarid shrimps, including only six hydrothermal vent decapods mitochondrial genomes (mitogenomes) [5]. On the basis of three mitochondrial genes and three nuclear genes, Sun et al (2018a) discovered that the most common recent ancestor of hydrothermal vent alvinocarid shrimps probably lived in the late Eocene or early Oligocene (~34.60 Ma) [9]. However, all these researches used either limited hydrothermal vent groups or a few DNA markers. The mitogenome contains increased phylogenetic signal and have widely used in the analysis of divergence history across a wide range of taxa from invertebrate [10–12] to vertebrate [13–17]. The mitogenomes have been shown to be useful for phylogenetic reconstruction and divergence time estimation in crustaceans [18–20]. At present, 18 mitogenomes of hydrothermal vent decapods are available (https://www.ncbi.nlm.nih.gov/), including 5 bythograeid crabs, 5 galatheid squat lobsters, and 8 alvinocarid shrimps. Unfortunately, the comparative analyses of the origin of this three hydrothermal vent groups based on mitogenomes still not been well addressed.

Mitochondrion is the main site of energy generation in cells, producing about 95% of the adenosine triphosphate (ATP) for the basic activities of life through oxidative phosphorylation (OXPHOS) [21–23]. Mitogenome encodes 13 essential OXPHOS proteins, which play an important part in the electron transport. Despite strong functional constraints, positive selection of mitochondrial DNA (mtDNA) may occurs when they exposed to environmental stress. Sometimes, the mitochondrial amino acid substitutions may be beneficial, underlying adaptive improvements in metabolic performance [24]. The mitochondrial gene polymorphisms has been considered relevant to the differences in thermal or metabolic needs among species, which either inhabited different environments [25–26] or employed distinct locomotive types [22, 27–28]. These findings have prompted scientists to examine the evolution of mitochondrial energy metabolism genes of the organisms living in challenging habitats [29]. Hydrothermal vents have been considered as one of the most extremely harsh environment on the Earth. Once the faunas invaded into hydrothermal vent habitats, they have to develop an adaptation mechanism to deal with the harsh and highly unstable conditions. In recent years, the advanced sequencing approaches allowed researchers to reveal how the hydrothermal vent animals have adapted to such inhospitable environments. However, most researchers focus their attention on the hydrothermal vent-associated transcription profile in vent macro-benthos, including mussels [30], worms [31] and shrimps [32–34]. In order to gain more knowledge about the genetic basis of organismal adaptation to hydrothermal vent environments, the positive selection signatures of mitochondrial genomes in the adaptation process are of particular interest.

The discovery of the different hydrothermal vent decapod groups lead to two questions. First, when did they invade hydrothermal vent and if the different vent organisms originated at the same time? Second, if there’re positive signatures among hydrothermal vent decapod mtDNA protein-coding genes. In other words, if the adoption of hydrothermal vent environment in decapods was associated with altered patterns of selection on mitochondrial function. To address these questions, we estimated the divergence time of the major lineages of Decapoda and reveal the origin of the hydrothermal vent bythograeid crabs, galatheid squat lobsters, and alvinocarid shrimps. Then we investigated the evolution of 13 mitochondrial protein coding genes to reveal the genetic basis for the adaptation of decapods to the hydrothermal vent environment.

## Materials and methods

### Source of data

67 complete mitogenomes of decapod crustaceans were used for our analysis, containing eighteen sequences of hydrothermal vent alvinocarid shrimps, bythograeid crabs and galatheid squat lobsters (S1 Table). Families of decapods, represented at least by one genus were included in the analysis, and when there are more than one species in the same genus, only one species is selected. The Alvinocardididae, Galatheoidea (except *Neopetrolisthes maculatus* and *Munida gregaria*) and Bythograeidae species selected in this study all inhabit hydrothermal vent condition.

### Phylogenetic analysis and divergence date estimation

The phylogenetic trees were reconstructed from 13 concatenated protein-coding genes with two methods. (i) Maximum Likelihood (ML) analyses was conducted using RAxML Black-Box webserver [35]. The node supports were evaluated by bootstrapping with 1000 replicates. Bayesian inference (BI) with Markov-chain Monte Carlo (MCMC) sampling was conducted using MrBayes 3.1 [36]. The MCMC was run for 10 million generations (sampling every 1000 generations) and the average SD of split frequencies was checked (<0.01). Convergence of sampled parameters were investigated in Tracer 1.6 [37] to ensure effective sampling size (ESS) for all parameters >200. The first 5,000 “burn-in” trees were omitted, and the remaining 5,000 sampled trees were used to estimate the 50% majority rule consensus trees. jModelTest [38] was used to select the best-fit nucleotide substitution models based on the Akaike Information Criterion (AIC).

Divergence times were estimated in BEAST 1.8.1 [39] using the random starting trees, the Yule speciation model, the relaxed uncorrelated lognormal clocks, and two runs of four MCMC chains each. The .xml input files for BEAST analyses were created in BEAUti 1.8.1 [39]. Samples from the posterior were drawn every 1000 generations over a total of 10 million generations every MCMC run. The software Tracer 1.5 [37] was used to assess the chain convergence by examining the ESS of parameters. TreeAnnotator 1.8.1 [39] was used to summarize the 95% highest posterior densities (HPD) and the maximum-clade-credibility tree topology with a 50% burn-in. The tree and divergence times were viewed using FigTree 1.4 [40].

We identified four fossil calibrations across Decapoda, justified based on previous practices [20, 41]. The fossil record for the Palaemonoidea has been found at early Cretaceous (99.6–112 Mya) based on the fossil species *Palaemon antonellae* [42] and the fossil record for the Atyoidae was found at early Cretaceous (124–127 Mya) based on *Delclosia martinelli* [43]. The age of Anomura and Astacidea were constrained at late Triassic (201.6–228 Mya) and middle Triassic (227–234 Mya), based on *Platykotta akaina* [44] and *Chimaerastacus pacifluvialis* [45], respectively. For fossil data, the earliest known representatives for the particular clades were chosen and the mean fossil ages were calculated. The occurrence of the fossil indicated the divergence of the ancestor prior to the fossil ages, so the fossil ages were applied as priors (lower bounds) to estimate the divergence time of the most recent common ancestry of their respective lineages.

### Selective pressure analyses

**Fig 1:**
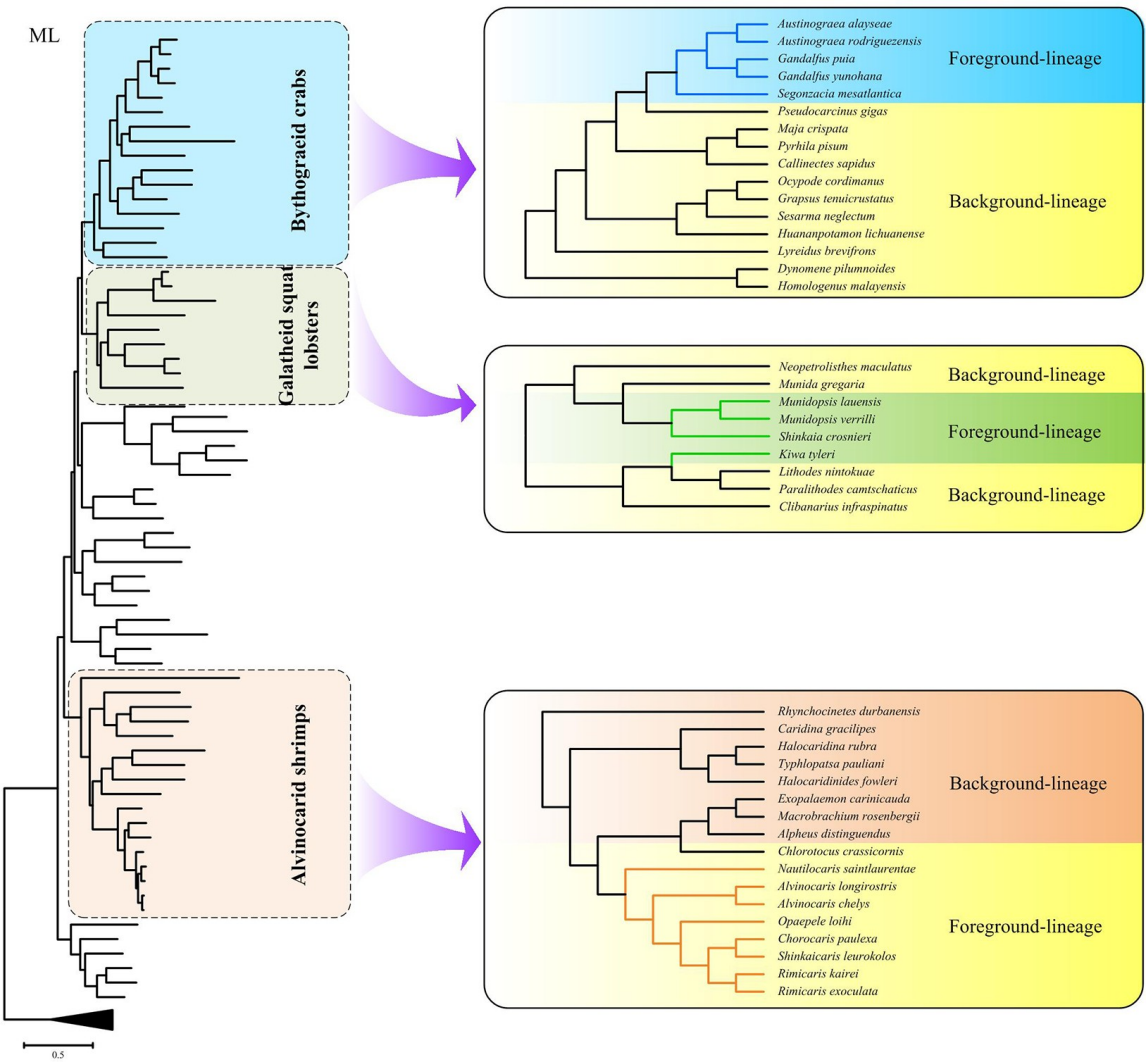
Phylogenetic trees used for selection analyses of mitochondrial OXPHOS genes in Bythograeidae, Galatheoidea and Alvinocarididae, respectively. The foreground-lineages were marked in blue (Bythograeidae), green (Galatheoidea) and orange (Alvinocarididae) and the background-lineages were marked in yellow.

CodeML implemented in the PAML package [46] was applied to evaluate the selective pressure in each of the 13 mitochondrial protein-coding genes, as well as the concatenated sequences. The nonsynonymous (Ka) to synonymous (Ks) rate ratio (Ka/Ks) measure the changes in selective pressures, where ω = 1, ω<1 and ω>1 indicates neutral evolution, purifying, and positive selection, respectively. As the selective pressure analyses is only informative when comparing sister taxa, we conducted the analyses using the subtrees of Bythograeidae, Galatheoidea and Alvinocarididae, respectively, which were built in this study (Fig 1).

The M0 model estimate the distribution of ɷ values as a benchmark under an assumption of no adaptive evolution in the gene sequences, which assumes a single ω ratio for all branches in the phylogeny. In order to test for positive selection, likelihood ratio tests (LRTs) were conducted by comparing the maximum likelihoods of a null model (M8a) against an alternative model (M8) [47].

Positive selection may act in very short episodes during the evolution of a protein [48], affecting only a few sites along lineages in the phylogeny. Here, the branch-site models [49] were used to test the positive selection of individual sites along foreground-lineages (hydrothermal vent decapod lineages), which allow selection pressure to vary both among sites in the protein and across branches on the tree. For this test, LRT were conducted to test whether the alternative model (MA) fits the data significantly better than the null model (MA0). The Bayes Empirical Bayes (BEB) method [50] was used to calculate posterior probabilities (> 0.90) to identify whether some sites have undergone positive selection in the foreground lineages among the phylogenetic tree.

In branch-site model, the branches of the phylogenetic tree were partitioned into foreground-(hydrothermal vent decapod lineages) and background-(the remaining branches) branches (Fig 1). Because the branch-site model do not allow for multiple foreground branches, we considered the branch leading to the hydrothermal vent bythograeid crabs, galatheid squat lobsters, and alvinocarid shrimps as foreground-lineage separately.

## Results

### Mitochondrial phylogeny

**Fig 2:**
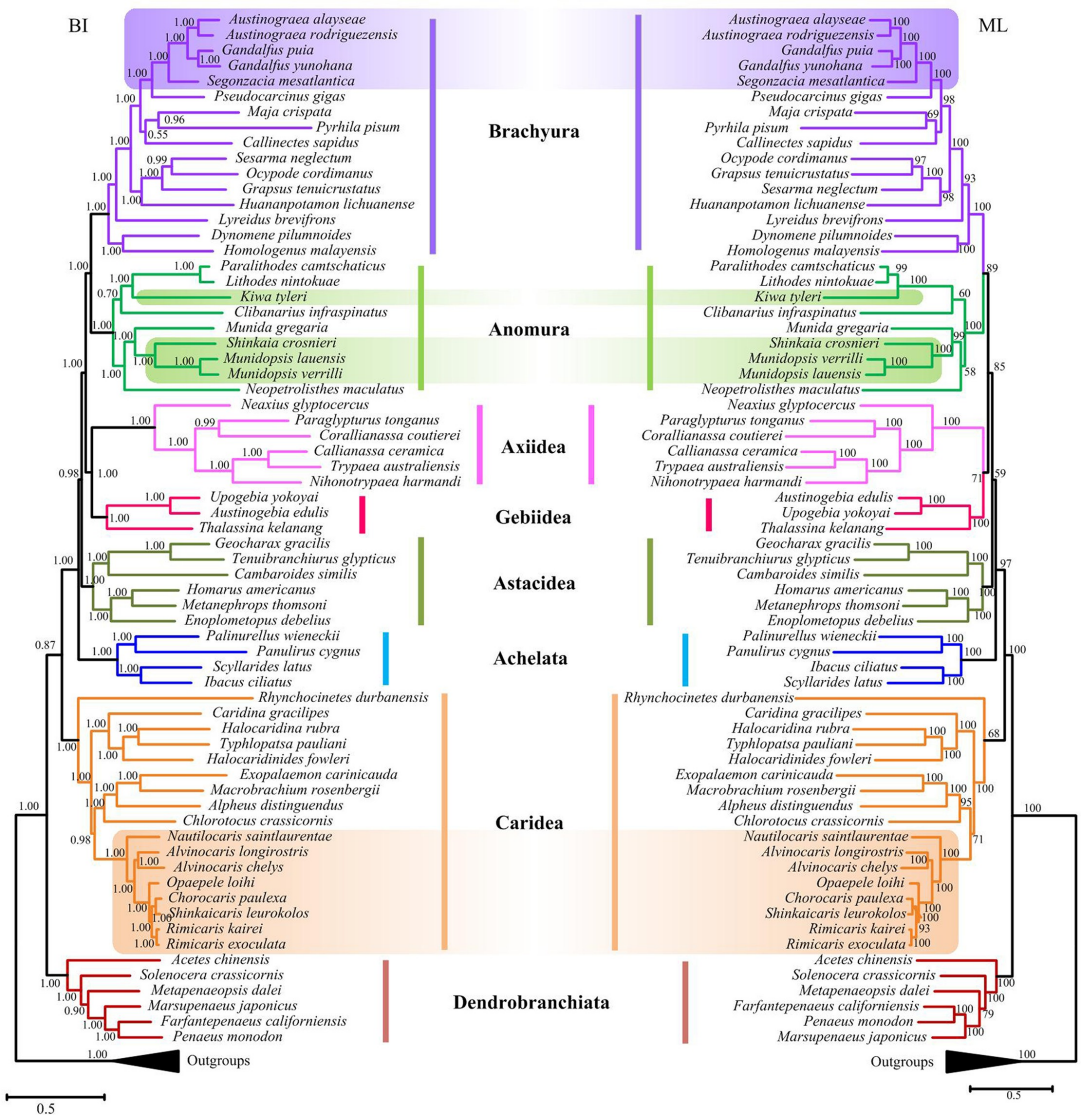
Phylogenetic trees of Decapoda derived from Maximum Likelihood (ML) and Bayesian analyses (BI) based on nucleotide sequences of 13 mitochondrial PCGs. The number at each node is the bootstrap probability (ML) and Bayesian posterior probability (BI). The purple, green and orange backgrounds indicate hydrothermal vent bythograeid crabs, galatheid squat lobsters and alvinocarid shrimps, respectively.

The molecular phylogeny of Decapoda was built based on the combined nucleotide sequences of 13 protein-coding genes using ML and BI methods. The tree topologies were congruent across different analytical methods, albeit with different levels of node support (Fig 2). Our study is not intended to test the monophyly of decapod infraorders, however, there is statistical support for all the infraorders. The species of Bythograeidae (hydrothermal vent crabs), Alvinocarididae (hydrothermal vent shrimps) and Galatheoidea (hydrothermal vent squat lobsters) were placed at more evolved positions in the trees. A sister relationship between the hydrothermal vent alvinocarid shrimps and Pandalidae + (Alpheidae + Palaemonidae) was recovered in the trees. The hydrothermal vent bythograeid crabs showed a sister position to Eriphiidae with strong nodal support (PP = 1.00/BP = 100%). However, the trees showed that the Galatheoidea (hydrothermal vent squat lobsters) do not cluster together, and the yeti crab *Kiwa tyleri* was more closely related to the lithodid crabs group *P*. *camtschaticus/L*. *nintokuae* (Fig 2).

### Divergence time of hydrothermal vent decapods

**Fig 3:**
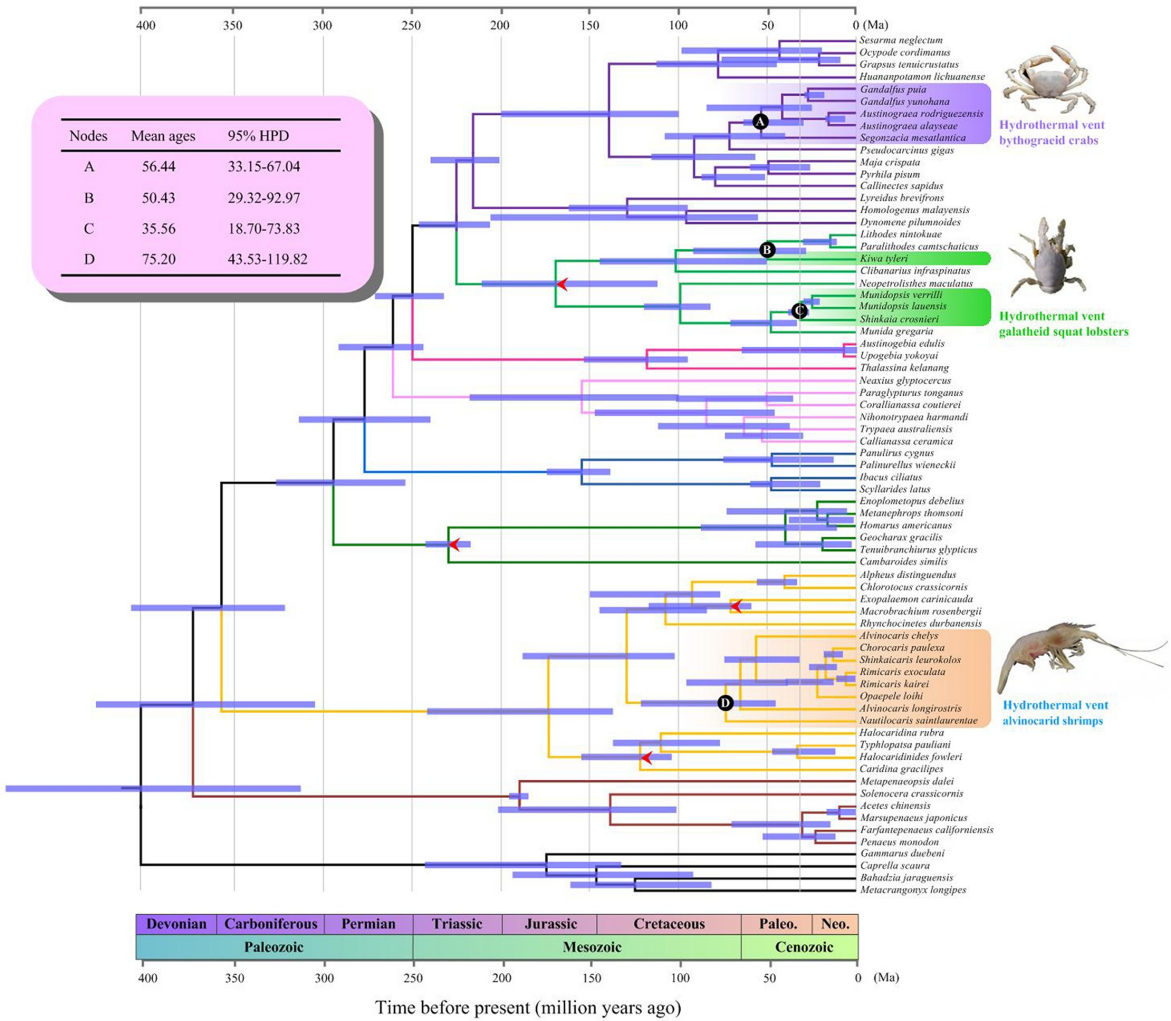
Estimates of divergence times using BEAST. Blue bars indicate 95% highest posterior density (HPD) intervals for nodes. The interested nodes are indicated by black circles, which correspond to the origin of hydrothermal vent bythograeid crabs (node A), galatheid squat lobsters (node B, C) and alvinocarid shrimps (node D). The mean divergence times and the 95% HPD of the interested nodes were shown on the top left corner. Species names and terminal branches are in purple, green and orange for hydrothermal vent bythograeid crabs, galatheid squat lobsters and alvinocarid shrimps, respectively. Calibrated nodes are indicated by arrows.

The posterior mean ages and the 95% HPD intervals are presented in Fig 3. The most recent common ancestor of hydrothermal vent alvinocarid shrimps was estimated at 75.20 Ma (95% HPD: 43.53–119.82 Ma) (Node A), which refers to the Late Cretaceous origin of alvinocarid shrimps. The divergence of hydrothermal vent bythograeid crabs occurred later, approximately 56.44 Ma (95% HPD: 33.15–67.04 Ma) (Node B). The divergence time estimates on the hydrothermal vent yeti crab *K*. *tyleri* and other galatheoid squat lobsters are located at 35.56 Ma (95% HPD: 18.70–73.83 Ma) (Node C) and 47.41 Ma (35.09–72.37 Ma) (Node D), respectively. Thus, the divergence time analysis refers to the Early Tertiary origin of hydrothermal vent bythograeid crabs and galatheoid squat lobsters.

### Analysis of selective pressure

#### Random-site analyses

We employed random-sites codon models to infer the longstanding patterns of molecular evolution of mitochondrial genes in Alvinocarididae, Bythograeidae and Galatheoidea datasets, respectively, analyzing both 13 individual protein-coding genes and a single concatenated sequence of all 13 genes (Conc). Fitting the M0 model resulted in an overall ω (Ka/Ks) estimate from 0.063 in Alvinocarididae group to 0.042 in Bythograeidae for the concatenated data set (Table 1). Gene-specific estimates of ω were less than 0.10 for all genes. *Atp8* showed the highest ω values (0.072–0.125) in all the three groups, while *cox1* showed the lowest evolutionary rates (0.011–0.018) (Table 1). The M8a-M8 test in the random-site model was applied to identify the site-specific positive selection. However, in no case did the test reveal evidence for positive selection (p-value > 0.05 for all tests; S2 Table). This also suggested that purifying selection was the dominant evolutionary force shaping decapod mitochondrial genes.

**Table 1:** The estimates of omega (ω), transition/transversion rate ratio (κ), and the number of synonymous substitutions per synonymous site (dS) in M0 model. The log-likelihood (lnL) is provided.

Groups	Gene	ω	κ	Branch-specific dS	lnL
				Estimated (Range)	
Alvinocarididae	*atp6*	0.041	2.520	0.03–6.03	-6377.64
	*atp8*	0.072	2.438	0.00–7.03	-1845.42
	*cox1*	0.018	3.156	0.02–2.08	-11150.66
	*cox2*	0.028	3.049	0.00–3.93	-5730.98
	*cox3*	0.026	2.445	0.01–3.26	-6221.97
	*cytb*	0.026	3.453	0.02–2.63	-9167.96
	*nad1*	0.021	2.582	0.05–3.99	-7709.96
	*nad2*	0.042	1.790	0.00–4.87	-11089.87
	*nad3*	0.054	2.423	0.00–2.63	-3404.52
	*nad4*	0.027	2.862	0.01–5.69	-13277.24
	*nad4l*	0.019	2.931	0.00–17.00	-2895.43
	*nad5*	0.027	2.287	0.04–7.07	-17284.97
	*nad6*	0.051	2.031	0.05–5.85	-6187.13
	Tconc	0.063	1.904	0.03–1.66	-106826.10
Bythograeidae	*atp6*	0.023	2.783	0.08–5.91	-5817.08
	*atp8*	0.125	1.411	0.00–3.57	-2000.27
	*cox1*	0.012	2.808	0.02–3.14	-10564.63
	*cox2*	0.025	2.706	0.20–5.34	-5594.81
	*cox3*	0.019	2.571	0.12–6.31	-6238.89
	*cytb*	0.023	3.294	0.14–4.14	-9368.52
	*nad1*	0.016	3.405	0.23–7.68	-7613.45
	*nad2*	0.048	1.648	0.00–6.65	-12390.04
	*nad3*	0.032	2.294	0.00–5.05	-3402.77
	*nad4*	0.028	2.819	0.31–5.34	-12804.60
	*nad4l*	0.021	3.153	0.00–9.49	-2806.88
	*nad5*	0.039	2.487	0.13–6.18	-17366.48
	*nad6*	0.034	1.738	0.34–9.42	-5678.57
	Tconc	0.042	2.091	0.22–3.64	-105762.57
Galatheoidea	*atp6*	0.042	2.533	0.07–3.18	-3860.33
	*atp8*	0.115	2.298	0.00–4.59	-1109.99
	*cox1*	0.011	3.609	0.24–2.76	-6654.78
	*cox2*	0.032	2.759	0.16–2.88	-3517.04
	*cox3*	0.030	2.213	0.11–2.14	-4042.18
	*cytb*	0.031	2.846	0.03–3.35	-6061.66
	*nad1*	0.020	3.217	0.19–6.56	-4869.72
	*nad2*	0.046	1.751	0.21–6.06	-6829.49
	*nad3*	0.028	2.521	0.00–5.38	-1981.91
	*nad4*	0.028	3.015	0.17–3.93	-7258.23
	*nad4l*	0.021	3.970	0.00–9.78	-1658.66
	*nad5*	0.034	1.925	0.29–4.11	-10182.82
	*nad6*	0.055	1.683	0.00–5.57	-3692.48
	Tconc	0.040	2.182	0.18–277	-63433.65

#### Branch-site analyses

In branch-site model, the BEB analyses (posterior probabilities (PP) > 95%) were used to identify the signatures of positive selection along the hydrothermal vent decapod branches. For Alvinocarididae, Bythograeidae and Galatheoidea, ω2 was estimated at 2.74, 2.38 and 15.33, respectively. In all the cases, the LRTs were highly significant (*p*-values < 0.001). BEB analysis of the concatenated data set of 13 PCGs identified 10 well-supported (PP ≥ 0.95) positively selected sites in three genes (*atp6*, *cox1*, *cytb*) when the Alvinocarididae branch was set as the foreground-lineage. Two genes (*nad2*, *nad4*) and two genes (*nad1*, *nad5*) were identified as positively selected when the Bythograeidae and Galatheoidea branches were set as the foreground-lineage, respectively (Table 2). The gene-specific BEB analysis identified the signature of positive selection in five genes along the Alvinocarididae branch (*atp6*, *cox1*, *cytb*, *nad3*, *nad4*), two genes along the Bythograeidae branch (*nad2*, *nad4*), and three genes along the Galatheoidea branches (*nad1*, *nad2*, *nad5*) (S3 Table). Although the discrepancies occurred between gene-specific and concatenated analyses, almost of the positively-selected sites identified through analysis of the concatenated data set were also found through gene-specific analyses, suggesting greater power for the gene-specific analyses. For both gene-specific and concatenated analyses, most of the positively selected sites were identified in CI proteins (47.06% and 41.67%, respectively), and to a lesser degree in CIII (29.41% and 33.33%, respectively) (Table 3).

**Table 2:** The positively selected sites of concatenated analyses (Conc) (branch-site model). Parameter estimates are shown only for the positive-selection site class (Site Class 2). Results of gene-specific analyses under the branch-site model are provided in S3 Table.

Branch	Branch-site	Site Class 2		lnL	2ΔL	*p*-value		Positively Selected Sites
	model	ω2	p2					Gene: site
Alvinocarididae	Model A	2.748	0.031	-102681.205			*atp6*	73 F 0.973*, 177 S 0.992**
	Null model	0.550	0.332	-102753.134	143.857	< 0.001	*cox1*	440 T 0.962*
							*cytb*	54 M 0.956*, 133 T 0.967*, 326 L 0.985*
Bythograeidae	Model A	2.377	0.010	-103780.683			*nad2*	234 L 0.970*
	Null model	0.004	0.658	-103846.144	130.922	< 0.001	*nad4*	377 V 0.967*
Galatheoidea	Model A	19.340	0.008	-62320.127			*nad1*	250 L 0.952*
	Null model	0.162	0.323	-62450.191	260.128	< 0.001	*nad5*	88 F 0.986*, 473 S 0.961*

* 0.95 < BEB < 0.99

** BEB < 0.99

**Table 3:** The distribution of positively selected sites among genes and OXPHOS complexes in branch-site model.

OXPHOS complex	Number of positively selected sites (Concatenated analysis)	Number of positively selected sites (Gene specific analyses)
CI	5	9
	*nad1* = 1 (0, 0, 1)	*nad1* = 1 (0, 0, 1)
	*nad2* = 1 (0, 1, 0)	*nad2* = 3 (0, 2, 1)
	*nad3* = 0	*nad3* = 1 (1, 0, 0)
	*nad4* = 1 (0, 1, 0)	*nad4* = 2 (1, 1, 0)
	*nad4l* = 0	*nad4l* = 0
	*nad5* = 2 (0, 0, 2)	*nad5* = 2 (0, 0, 2)
	*nad6* = 0	*nad6* = 0
CIII	3	4
	*cytb* = 3 (3, 0, 0)	*cytb* = 4 (4, 0, 0)
CIV	1	2
	*cox1* = 1 (1, 0, 0)	*cox1* = 2 (2, 0, 0)
	*cox2* = 0	*cox2* = 0
	*cox3* = 0	*cox3* = 0
CV	2	3
	*atp6* = 2 (3, 0, 0)	*atp6* = 3 (3, 0, 0)
	*atp8* = 0	*atp8* = 0

## Discussion

Mitochondrial genes have been widely used in studies of phylogeny [51–53], phylogeography [54–55] and evolution [25, 56–57]. In this study, 67 mitogenomes were used to evaluate the phylogenetic position of the hydrothermal vent bythograeid crabs, galatheid squat lobsters, and alvinocarid shrimps, particularly focusing on the origin of the hydrothermal vent species.

Our phylogenetic analyses based on the nucleotide sequences of 13 protein-coding genes with two analytical methods recovered a highly supported phylogeny of Decapoda, albeit with different levels of support. The results were consistent with that of our previous study [20]. In this context, our phylogenetic analyses showed that hydrothermal vent bythograeid crabs, galatheid squat lobsters, and alvinocarid shrimps were located at more evolved positions in the trees. These observations suggest that they migrated from non-vent environments, instead of the remnants of ancient hydrothermal vent species, which support the extinction/repopulation hypothesis [7].

The divergence time estimation is greatly helpful for understanding the origin and evolutionary history of hydrothermal vent decapods. The age estimation for alvinocarid shrimps is slightly old than the timeline presented by Yang et al. (2013) who estimated alvinocarid shrimps origins in the Late Cretaceous/Early Tertiary, 51.5–69.7 Ma [5]. However, this new timescale indicates a much earlier time than the Early Oligocene origin, which estimated in our previous study where three mitochondrial genes and three nuclear genes were used [9]. This discrepancy may due to the heterogeneity of data. Our divergence time estimation for bythograeid crabs is congruent with the timeline illustrated by Yang et al. (2013), which also refers to the Early Tertiary origin of bythograeid crabs, 51.5–69.7 Ma [5]. In this study, we first dated the divergence time of hydrothermal vent galatheid squat lobsters. Although they are polyphyletic, the origin of the yeti crab *K*. *tyleri* closely corresponded with other galatheoid squat lobsters', suggesting the Early Tertiary divergence of galatheid squat lobsters. Our phylogenetic chronogram postulates recent diversification times for the hydrothermal vent alvinocarid shrimps, bythograeid crabs and galatheid squat lobsters. It is interesting to note that the Late Cretaceous and Early Tertiary boundary was characterized by relatively frequent anoxic/dysoxic bottom conditions in deep-sea habitats, which are thought to lead a massive extinctions of nearly all contemporary vent species [7]. During this period, some smaller-scale extinction events linked to anoxic events, ocean acidification and dramatic environmental changes also occurred in regional ocean [7, 58–59]. After these mass extinctions, the vent habitats may be recolonized by deep-sea or shallow-water ancestors. Some other modern vent taxa, such as deep-sea gastropods, vestimentiferan tubeworms, vesicomyid clams, bathymodiolid mussels also evolved recently (≤100 Ma), suggesting that they may must be derived from surrounding non-hydrothermal vent habitats [1, 3]. The recent diversification of the modern vent invertebrate species provided evidences for the extinction/repopulation hypothesis.

We tested for selective evolution in the mitochondrial PCGs of decapods in relation to hydrothermal vent adaptation, reasoning that the extremely harsh hydrothermal vent environment may have impacted the evolution of the mitochondrial-encoded OXPHOS proteins. Random-site models revealed that 13 mitochondrial PCGs of hydrothermal vent decapod species were all under negative constraints (purifying selection) (ω < 1), although the degree of selective constraint varied among genes. This was confirmed through the LRT analyses of M8a-M8, reinforcing the crucial and conserved OXPHOS functions of mtDNA protein-coding genes in decapods, as the general trend in eukaryotes [60]. That’s said, positive selection may have operated intermittently. The branch-site analyses confirmed this notion, with strong signatures of positive selection detected along the lineages leading to hydrothermal vent decapods.

Our investigation indicated that the convergent habitat selection in Alvinocarididae, Bythograeidae and Galatheoidea lineages may have been accompanied by convergent molecular evolution of the mitochondrial OXPHOS genes. Although no overlap was found among the lists of positively selected sites in the three hydrothermal vent lineages, the concordant complex scale was observed, suggesting that the convergence may express at a higher functional level, such as the OXPHOS complex level. For the three hydrothermal vent lineages, we found that most of the positively selected sites were located in Complex I (NADH dehydrogenase), whether for the concatenated or gene-specific analyses. This finding was in agreement with the reports of a recent meta-analysis of natural selection in metazoans mitochondria, which discovered that Complex I was the repeated target of positive selection in diverse taxa [61]. Perhaps it was because that Complex I is the first and largest enzyme complex in the mitochondrial OXPHOS pathway [25, 62], producing about 40% of the proton flux that drives ATP synthase [63]. The NADH dehydrogenase genes have been considered important in the adaptive evolution of the deep-sea anemone [64] and alvinocaridid shrimps [65]. In addition, NADH dehydrogenase genes were also detected to be under positive selection pressure in high altitude Tibetan horses [66–67], Chinese snub-nosed monkeys [68] and plateau galliform birds [69], providing evidences of Complex I in high-altitude adaptation. These observations, combined with ours, highlight the functions of Complex I as a proton pump, and improve our understanding that Complex I is an essential domain in the adaptation of decapods to the hydrothermal vent environments.

Positive selection were also detected outside of Complex I and they may also influence critical steps of mitochondrial electron transport chain. Positively selected sites were identified within *atp6*, *cox1* and *cytb* genes along the Alvinocarididae branch, when considering both the concatenated and gene-specific analyses. ATP synthase is the last enzyme complex in the OXPHOS system, and it is directly associated with the produce of ATP [64, 69–70]. The adaptive evolution of the ATPase genes could improve the adaptation to different environments [25, 64, 66, 71]. Complexes III (cytochrome *bc*_1_) and IV (cytochrome *c* oxidase) use direct coupling for electron transfer and proton translocation, which is mediated by membrane-embedded cofactors (haems and metal centres) [72]. Earlier studies have revealed signatures of adaptive evolution in the cytochrome c oxidase genes of Tibetan antelope [73], plateau pika [74] and camelid [75]. The cytochrome *b* gene of alpacas [25] may have experienced positive selection during adaptation to high-altitude. All these studies strengthen our view of the adaptive evolution of Complexes III and IV in the organisms living in the extreme environments.

## Conclusion

In this study, we used eighteen hydrothermal vent decapod mitogenomes to explore the evolutionary history and their adaptive evolution to the hydrothermal vent environment. Phylogenetic analysis supported that the deep-sea hydrothermal vent alvinocarid shrimps, galatheid squat lobsters, and bythograeid crabs may originated from surrounding non-hydrothermal vent habitats. Estimates of divergence time showed that the three hydrothermal vent decapod groups evolved recently, most likely invading in vent habitats during the Late Cretaceous and Early Tertiary. This may associate with the global deep-water anoxic/dysoxic events during this period. The mitochondrial PCGs showed an accelerated evolutionary rate in the hydrothermal vent decapod species, which may be beneficial for their adaptation to hydrothermal vent habitats. Total eleven positively selected sites were detected in the mitochondrial OXPHOS genes of three lineages of hydrothermal vent decapods, suggesting a link between hydrothermal vent adaption and OXPHOS molecular biology in decapods. The present investigation strengthen our view of adaptive evolution in the mitogenome of hydrothermal vent decapods.

## Supporting information

S1 Table List of samples and their corresponding accession numbers included in the analyses in this study. (PDF)

S2 Table M8a-M8 model analyses for each of the mitochondrial PCGs and the concatenated data sets. (PDF)

S3 Table Branch-site model analyses of the gene-specific mitochondrial data sets. (PDF)
