# Supplementary material for: Divergence history and hydrothermal vent adaptation of decapod crustaceans: A mitogenomic perspective
Source: PLoS One. 2019 Oct 29;14(10):e0224373. doi: 10.1371/journal.pone.0224373 (PMC6818795; doi:10.1371/journal.pone.0224373)
Supplement: S1 Table — (PDF) [file pone.0224373.s001.pdf]

S1 Table List of samples and their corresponding accession numbers included in the analyses in this study.

Infraorder	Species	GenBank NO.	
Achelata	*Scyllarides latus*	KC107814	
	Ibacus ciliatus	KM488334	
	Palinurellus wieneckii	KC847078	
	Panulirus cygnus	KT696496	
Anomura	*Paralithodes camtschaticus*	JX944381	
	Lithodes nintokuae	AB769476	
	Clibanarius infraspinatus	LN626968	
	Kiwa tyleri	KY423514	Hydrothermal vent
	Munida gregaria	KU521508	
	Neopetrolisthes maculatus	KC107816	
	Shinkaia crosnieri	EU420129	Hydrothermal vent
	Munidopsis lauensis	MH717895	Hydrothermal vent
	Munidopsis verrilli	MH717896	Hydrothermal vent
Astacidea	*Geocharax gracilis*	HG942174	
	Tenuibranchiurus glypticus	KM453741	
	Enoplometopus debelius	KM488333	
	Homarus americanus	HQ402925	
	Metanephrops thomsoni	KP889215	
	Cambaroides similis	JN991196	
Axiidea	*Neaxius glyptocercus*	JN897379	
	Callianassa ceramica	KU350630	
	Corallianassa coutierei	KC107817	
	Nihonotrypaea harmandi	LC221567	
	Paraglypturus tonganus	KJ820739	
	Trypaea australiensis	KM501040	
Caridea	*Alpheus distinguendus*	GQ892049	
	Caridina gracilipes	KM023648	
	Halocaridina rubra	DQ917432	
	Halocaridinides fowleri	KX844723	
	Typhlopatsa pauliani	KX844716	
	Alvinocaris chelys	JX184903	Hydrothermal vent
	Alvinocaris longirostris	JQ035659	Hydrothermal vent
	Nautilocaris saintlaurentae	KF226726	Hydrothermal vent
	Opaepele loihi	JQ035657	Hydrothermal vent
	Rimicaris exoculata	KP284529	Hydrothermal vent
	Rimicaris kairei	JQ035656	Hydrothermal vent
	Shinkaicaris leurokolos	MF627741	Hydrothermal vent
	Chorocaris paulexa	MK309612	Hydrothermal vent
	Chlorotocus crassicornis	KY944589	

	Exopalaemon carinicauda	EF560650	
	Macrobrachium rosenbergii	AY659990	
	Rhynchocinetes durbanensis	KT590405	
Gebiidea	*Thalassina kelanang*	JN897378	
	Upogebia yokoyai	KM886610	
	Austinogebia edulis	JN897376	
Brachyura	*Dynomene pilumnoides*	KT182070	
	Homologenus malayensis	KJ612407	
	Austinograea alayseae	JQ035660	Hydrothermal vent
	Austinograea rodriguezensis	JQ035658	Hydrothermal vent
	Segonzacia mesatlantica	KY541839	Hydrothermal vent
	Gandalfus puia	KR002727	Hydrothermal vent
	Gandalfus yunohana	EU647222	Hydrothermal vent
	Callinectes sapidus	AY363392	
	Sesarma neglectum	KX156954	
	Grapsus tenuicrustatus	KT878721	
	Huananpotamon lichuanense	KX639824	
	Maja crispata	KY650651	
	Lyreidus brevifrons	KM983394	
	Ocypode cordimanus	KT896743	
	Pseudocarcinus gigas	AY562127	
	Pyrhila pisum	KU343210	
Dendrobranchiata	*Farfantepenaeus californiensis*	EU497054	
	Marsupenaeus japonicus	KY363851	
	Metapenaeopsis dalei	KU050082	
	Penaeus monodon	AF217843	
	Solenocera crassicornis	KU899137	
	Acetes chinensis	JN689221	
Outgroup	*Gammarus duebeni*	JN704067	
	Bahadzia jaraguensis	FR872382	
	Metacrangonyx longipes	AM944817	
	Caprella scaura	AB539699	
