# Supplementary material for: Divergence history and hydrothermal vent adaptation of decapod crustaceans: A mitogenomic perspective
Source: PLoS One. 2019 Oct 29;14(10):e0224373. doi: 10.1371/journal.pone.0224373 (PMC6818795; doi:10.1371/journal.pone.0224373)
Supplement: S2 Table — (PDF) [file pone.0224373.s002.pdf]

S2 Table Random-site M8a M8 model analyses for each of the mitochondrial PCGs and the concatenated data sets.

Groups	Genes	Model	lnL	Parameter estimates	LRT	*p-value*
Alvinocarididae	ATP6	M8a	-6209.9125	p0 = 0.99131 p = 0.44530 q = 8.69500 (p1 = 0.00869) ω = 1.00000	1.884	>0.05
		M8	-6210.8546	p0 = 0.99993 p = 0.42048 q = 6.45259 (p1 = 0.01007) ω = 1.00000		
	ATP8	M8a	-1761.3322	p0 = 0.99983 p = 0.50816 q = 5.66988 (p1 = 0.02017) ω = 1.00000	0.051	>0.05
		M8	-1761.3575	p0 = 0.99422 p = 0.41959 q = 3.81885 (p1 = 0.01578) ω = 2.14059		
	COX1	M8a	-10962.0609	p0 = 0.99999 p = 0.19955 q = 9.35680 (p1 = 0.00001) ω = 1.00000	1.916	>0.05
		M8	-10963.0190	p0 = 0.99999 p = 0.02272 q = 0.17439 (p1 = 0.00001) ω = 3.74663		
	COX2	M8a	-5583.9895	p0 = 0.99999 p = 0.38674 q = 11.20195 (p1 = 0.00001) ω = 1.00000	1.351	>0.05
		M8	-5583.3139	p0 = 0.99999 p = 0.00735 q = 0.04203 (p1 = 0.01492) ω = 9.03886		
	COX3	M8a	-6070.0502	p0 = 0.99654 p = 0.30189 q = 9.62144 (p1 = 0.00346) ω = 1.00000	1.215	>0.05
		M8	-6070.6579	p0 = 0.99622 p = 0.29457 q = 6.48133 (p1 = 0.00378) ω = 2.94105		
	CYTB	M8a	-8954.5739	p0 = 0.99999 p = 0.28908 q = 9.48523 (p1 = 0.00001) ω = 1.00000	0.177	>0.05
		M8	-8954.6626	p0 = 0.99999 p = 0.01479 q = 0.09568 (p1 = 0.00001) ω = 7.31221		
	ND1	M8a	-7436.1360	p0 = 0.99999 p = 0.27080 q = 8.46251 (p1 = 0.00001) ω = 1.00000	0.320	>0.05
		M8	-7436.2958	p0 = 0.99999 p = 0.30873 q = 7.31364 (p1 = 0.00001) ω = 1.00000		
	ND2	M8a	-10712.3099	p0 = 0.99670 p = 0.52800 q = 10.96826 (p1 = 0.00330) ω = 1.00000	0.396	>0.05
		M8	-10712.5078	p0 = 0.98723 p = 0.55767 q = 10.37092 (p1 = 0.01277) ω = 1.00000		
	ND3	M8a	-3243.0483	p0 = 0.99999 p = 0.28600 q = 3.57033 (p1 = 0.00001) ω = 1.00000	1.633	>0.05
		M8	-3242.2317	p0 = 0.98878 p = 0.28734 q = 3.25962 (p1 = 0.01122) ω = 1.95103		
	ND4	M8a	-12716.1489	p0 = 0.99877 p = 0.35511 q = 11.64177 (p1 = 0.00123) ω = 1.00000	1.436	>0.05
		M8	-12716.8670	p0 = 0.99751 p = 0.37350 q = 9.36515 (p1 = 0.00249) ω = 1.00000		
	ND4L	M8a	-2775.6774	p0 = 0.99999 p = 0.43883 q = 8.98739 (p1 = 0.00001) ω = 1.00000	0.811	>0.05
		M8	-2775.2720	p0 = 0.99999 p = 0.35629 q = 15.67295 (p1 = 0.00001) ω = 1.00000		
	ND5	M8a	-16471.1711	p0 = 0.99562 p = 0.32683 q = 9.60473 (p1 = 0.00438) ω = 1.00000	0.457	>0.05
		M8	-16471.3993	p0 = 0.99455 p = 0.37738 q = 7.80942 (p1 = 0.00545) ω = 1.54822		
	ND6	M8a	-5936.7870	p0 = 0.97645 p = 0.47125 q = 7.19647 (p1 = 0.02355) ω = 1.00000	0.002	>0.05
		M8	-5936.7862	p0 = 0.96954 p = 0.50602 q = 7.09582 (p1 = 0.03046) ω = 1.00000		

	Tconc	M8a	-102640.2029	p0 = 0.99147 p = 0.30596 q = 3.43704 (p1 = 0.00853) ω = 1.00000	1.093	>0.05
		M8	-102640.7495	p0 = 0.97513 p = 0.33864 q = 3.28210 (p1 = 0.02487) ω = 6.56589		
Bythograeidae	ATP6	M8a	-5599.0775	p0 = 0.99999 p = 0.23242 q = 6.33379 (p1 = 0.00001) ω = 1.00000	0.212	>0.05
		M8	-5599.1836	p0 = 0.98495 p = 0.25959 q = 6.60566 (p1 = 0.01505) ω = 1.00000		
	ATP8	M8a	-1899.4242	p0 = 0.99999 p = 0.63348 q = 2.44504 (p1 = 0.00001) ω = 1.00000	0.431	>0.05
		M8	-1899.2084	p0 = 0.99999 p = 0.50242 q = 2.02638 (p1 = 0.00001) ω = 1.00000		
	COX1	M8a	-10376.6207	p0 = 0.99805 p = 0.15321 q = 9.30280 (p1 = 0.00195) ω = 1.00000	1.271	>0.05
		M8	-10377.2564	p0 = 0.99787 p = 0.15147 q = 5.76322 (p1 = 0.00213) ω = 5.16760		
	COX2	M8a	-5439.2952	p0 = 0.99999 p = 0.31952 q = 10.19837 (p1 = 0.00001) ω = 1.00000	1.230	>0.05
		M8	-5438.6801	p0 = 0.99999 p = 0.32615 q = 7.99735 (p1 = 0.00001) ω = 9.87278		
	COX3	M8a	-6057.7687	p0 = 0.99999 p = 0.21696 q = 8.65639 (p1 = 0.00001) ω = 1.00000	2.201	>0.05
		M8	-6058.8692	p0 = 0.99999 p = 0.24477 q = 6.58516 (p1 = 0.00001) ω = 2.80409		
	CYTB	M8a	-9068.2257	p0 = 0.99490 p = 0.27292 q = 9.54815 (p1 = 0.00510) ω = 1.00000	1.029	>0.05
		M8	-9068.7400	p0 = 0.99214 p = 0.28596 q = 7.75522 (p1 = 0.00786) ω = 3.80545		
	ND1	M8a	-7315.1035	p0 = 0.99999 p = 0.26730 q = 11.62444 (p1 = 0.00001) ω = 1.00000	1.622	>0.05
		M8	-7315.9144	p0 = 0.99999 p = 0.31185 q = 9.71465 (p1 = 0.00001) ω = 6.30598		
	ND2	M8a	-11974.6502	p0 = 0.99999 p = 0.69902 q = 10.49418 (p1 = 0.00001) ω = 1.00000	0.446	>0.05
		M8	-11974.4275	p0 = 0.99242 p = 0.65318 q = 8.99923 (p1 = 0.00758) ω = 1.00000		
	ND3	M8a	-3240.7785	p0 = 0.96772 p = 0.32226 q = 9.46505 (p1 = 0.03228) ω = 1.00000	0.020	>0.05
		M8	-3240.7884	p0 = 0.96384 p = 0.34067 q = 7.29881 (p1 = 0.03616) ω = 1.00000		
	ND4	M8a	-12265.5086	p0 = 0.99999 p = 0.37704 q = 9.52708 (p1 = 0.00001) ω = 1.00000	1.589	>0.05
		M8	-12266.3030	p0 = 0.99696 p = 0.40678 q = 8.00769 (p1 = 0.00304) ω = 1.00000		
	ND4L	M8a	-2672.5252	p0 = 0.99999 p = 0.34678 q = 10.56793 (p1 = 0.00001) ω = 1.00000	1.168	>0.05
		M8	-2673.1093	p0 = 0.99999 p = 0.42060 q = 9.77297 (p1 = 0.00001) ω = 9.20977		
	ND5	M8a	-16504.6212	p0 = 0.99519 p = 0.35534 q = 7.02762 (p1 = 0.00481) ω = 1.00000	1.592	>0.05
		M8	-16505.4172	p0 = 0.99609 p = 0.39753 q = 6.72491 (p1 = 0.01391) ω = 1.54822		
	ND6	M8a	-5391.4143	p0 = 0.99999 p = 0.39403 q = 6.55504 (p1 = 0.00621) ω = 1.00000	1.873	>0.05
		M8	-5392.3506	p0 = 0.99999 p = 0.45727 q = 4.53167 (p1 = 0.00505) ω = 4.55317		
	Tconc	M8a	-100877.2627	p0 = 0.99460 p = 0.27824 q = 4.56501 (p1 = 0.00540) ω = 1.00000	0.722	>0.05
		M8	-100877.6238	p0 = 0.99298 p = 0.31645 q = 4.29184 (p1 = 0.01702) ω = 1.00000		

Galatheoidea	ATP6	M8a	-3760.7819	p0 = 0.99533 p = 0.35677 q = 6.25384 (p1 = 0.00001) ω = 1.00000	0.816	>0.05
		M8	-3761.1897	p0 = 0.99999 p = 0.31580 q = 4.55514 (p1 = 0.00001) ω = 1.00000		
	ATP8	M8a	-1043.9403	p0 = 0.99999 p = 0.53258 q = 4.02335 (p1 = 0.00001) ω = 1.00000	0.283	>0.05
		M8	-1044.0818	p0 = 0.92132 p = 0.32625 q = 1.55336 (p1 = 0.07868) ω = 3.64758		
	COX1	M8a	-6578.0859	p0 = 0.99780 p = 0.22223 q = 16.43980 (p1 = 0.00220) ω = 1.00000	0.683	>0.05
		M8	-6578.4272	p0 = 0.99713 p = 0.03443 q = 0.40272 (p1 = 0.00287) ω = 1.00000		
	COX2	M8a	-3424.6364	p0 = 0.98594 p = 0.37210 q = 11.16839 (p1 = 0.01406) ω = 1.00000	0.324	>0.05
		M8	-3424.7984	p0 = 0.98378 p = 0.30630 q = 7.46984 (p1 = 0.01622) ω = 1.00000		
	COX3	M8a	-3927.1410	p0 = 0.99999 p = 0.21924 q = 5.04219 (p1 = 0.00001) ω = 1.00000	1.639	>0.05
		M8	-3927.9602	p0 = 0.99999 p = 0.22771 q = 3.68398 (p1 = 0.00001) ω = 1.00000		
	CYTB	M8a	-5900.2595	p0 = 0.99999 p = 0.32527 q = 7.98546 (p1 = 0.00001) ω = 1.00000	1.299	>0.05
		M8	-5900.9087	p0 = 0.99714 p = 0.30531 q = 6.08773 (p1 = 0.00286) ω = 3.80545		
	ND1	M8a	-4755.0887	p0 = 0.99999 p = 0.37126 q = 12.80997 (p1 = 0.00001) ω = 1.00000	1.642	>0.05
		M8	-4755.9099	p0 = 0.99924 p = 0.35041 q = 10.15072 (p1 = 0.00076) ω = 4.26138		
	ND2	M8a	-6619.8712	p0 = 0.99410 p = 0.53481 q = 8.50173 (p1 = 0.00590) ω = 1.00000	1.580	>0.05
		M8	-6620.6613	p0 = 0.99999 p = 0.65068 q = 11.77773 (p1 = 0.00001) ω = 1.00000		
	ND3	M8a	-1905.4806	p0 = 0.99149 p = 0.49628 q = 7.25865 (p1 = 0.00851) ω = 1.00000	0.272	>0.05
		M8	-1905.6167	p0 = 0.99999 p = 0.30466 q = 5.17631 (p1 = 0.00001) ω = 7.50652		
	ND4	M8a	-7041.2502	p0 = 0.99999 p = 0.32912 q = 8.40756 (p1 = 0.00001) ω = 1.00000	1.245	>0.05
		M8	-7041.8725	p0 = 0.99999 p = 0.32339 q = 6.46011 (p1 = 0.00001) ω = 7.90944		
	ND4L	M8a	-1593.3767	p0 = 0.99999 p = 0.34199 q = 12.15540 (p1 = 0.00001) ω = 1.00000	0.663	>0.05
		M8	-1593.7082	p0 = 0.99999 p = 0.33249 q = 9.12755 (p1 = 0.00001) ω = 1.95279		
	ND5	M8a	-9731.1592	p0 = 0.99625 p = 0.29296 q = 5.67125 (p1 = 0.00375) ω = 1.00000	0.110	>0.05
		M8	-9731.1041	p0 = 0.99208 p = 0.32295 q = 4.86319 (p1 = 0.00792) ω = 1.54822		
	ND6	M8a	-3563.8774	p0 = 0.99391 p = 0.53127 q = 6.74405 (p1 = 0.01609) ω = 1.00000	0.605	>0.05
		M8	-3564.1798	p0 = 0.99289 p = 0.45997 q = 4.33851 (p1 = 0.00711) ω = 1.78591		
	Tconc	M8a	-61220.2644	p0 = 0.99565 p = 0.29434 q = 4.99432 (p1 = 0.00435) ω = 1.00000	0.189	>0.05
		M8	-61220.3589	p0 = 0.99354 p = 0.30248 q = 4.51980 (p1 = 0.01646) ω = 1.00000		
