# Supplementary material for: Divergence history and hydrothermal vent adaptation of decapod crustaceans: A mitogenomic perspective
Source: PLoS One. 2019 Oct 29;14(10):e0224373. doi: 10.1371/journal.pone.0224373 (PMC6818795; doi:10.1371/journal.pone.0224373)
Supplement: S3 Table — (PDF) [file pone.0224373.s003.pdf]

S3 Table Branch-site model analyses of the gene-specific mitochondrial data sets.

Branch	Genes	BrS model (n.p.)	Site Class 0		Site Class1		Site Class 2		lnL	LRT	PSS (BEB PP)
			ω0	*p0*	ω1	p_1	ω2	p_2			
Alvinocaridida e	ATP6	Model A	0.03174	0.91011	1.00000	0.06355	9.86181	0.02461	-6312.0818	5.8554	15 N 0.985*, 73 F 0.973*, 177 S 0.967*
		Null model	0.03123	0.89802	1.00000	0.06316	1.00000	0.03627	-6315.0095		
	ATP8	Model A	0.06036	0.09729	1.00000	0.03509	4.97823	0.63766	-1797.4136	0.0001	
		Null model	0.06036	0.64373	1.00000	0.23215	1.00000	0.09122	-1797.4136		
	COX1	Model A	0.01659	0.93209	1.00000	0.00641	1.14781	0.06108	-11132.0510	4.0258	149 V 0.985*, 440 T 0.973*
		Null model	0.01659	0.92767	1.00000	0.00638	1.00000	0.06551	-11134.0639		
	COX2	Model A	0.02632	0.75425	1.00000	0.02706	1.00000	0.21112	-5710.5107	0.0000	
		Null model	0.02632	0.75425	1.00000	0.02706	1.00000	0.21112	-5710.5107		
	COX3	Model A	0.02413	0.97924	1.00000	0.01035	28.22091	0.01030	-6206.7545	1.4883	
		Null model	0.02444	0.98985	1.00000	0.01015	1.00000	0.00000	-6207.4986		
	CYTB	Model A	0.02305	0.91790	1.00000	0.01887	1.09796	0.06195	-9146.2170	0.0254	54 M 0.956*, 133 T 0.967*, 253 M 0.973*, 326 L 0.985*
		Null model	0.02302	0.91582	1.00000	0.01878	1.00000	0.06408	-9146.2297		
	ND1	Model A	0.01817	0.91453	1.00000	0.05231	5.73564	0.03136	-7641.9487	5.5805	
		Null model	0.01833	0.90679	1.00000	0.05190	1.00000	0.03908	-7644.7389		
	ND2	Model A	0.03876	0.84205	1.00000	0.09066	1.00000	0.06075	-10973.2493	0.0000	
		Null model	0.03876	0.84205	1.00000	0.09066	1.00000	0.06075	-10973.2493		
	ND3	Model A	0.03421	0.75566	1.00000	0.21705	998.99880	0.02120	-3344.6890	1.7144	43 G 0.988*
		Null model	0.03195	0.74102	1.00000	0.24290	1.00000	0.01211	-3345.5462		
	ND4	Model A	0.03332	0.87022	1.00000	0.11040	42.14510	0.01720	-13142.4019	2.4007	141 S 0.975*
		Null model	0.03303	0.86439	1.00000	0.11064	1.00000	0.02213	-13143.6023		
	ND4L	Model A	0.03352	0.48853	1.00000	0.12747	1.00000	0.30454	-2890.5459	0.0000	
		Null model	0.03352	0.48854	1.00000	0.12747	1.00000	0.30453	-2890.5459		

	ND5	Model A	0.02991	0.86736	1.00000	0.13264	10.39820	0.00000	-16987.6431	0.0007	
		Null model	0.02991	0.86736	1.00000	0.13264	1.00000	0.00000	-16987.6435		
	ND6	Model A	0.03441	0.69317	1.00000	0.30683	1.00000	0.00000	-6086.6708	0.0000	
		Null model	0.03441	0.69317	1.00000	0.30683	1.00000	0.00000	-6086.6708		
Bythograeidae	ATP6	Model A	0.01857	0.16762	1.00000	0.01057	6.19063	0.77307	-5751.5976	1.3503	
		Null model	0.01868	0.94095	1.00000	0.05905	1.00000	0.00000	-5752.2728		
	ATP8	Model A	0.09534	0.56976	1.00000	0.43024	1.00000	0.00000	-1929.3312	0.0000	
		Null model	0.09534	0.56976	1.00000	0.43024	1.00000	0.00000	-1929.3312		
	COX1	Model A	0.01015	0.00000	1.00000	0.00000	62.56968	0.99070	-10524.1121	0.0974	
		Null model	0.01015	0.00000	1.00000	0.00000	1.00000	0.99070	-10524.0634		
	COX2	Model A	0.02388	0.95622	1.00000	0.01900	1.00000	0.02429	-5574.8340	0.0000	
		Null model	0.02388	0.95622	1.00000	0.01900	1.00000	0.02429	-5574.8340		
	COX3	Model A	0.01683	0.97256	1.00000	0.02030	2.33623	0.00699	-6218.9231	0.6174	
		Null model	0.01708	0.97944	1.00000	0.02056	1.00000	0.00000	-6219.2318		
	CYTB	Model A	0.02044	0.95984	1.00000	0.02842	1.21305	0.01139	-9284.8125	0.0168	
		Null model	0.02044	0.95821	1.00000	0.02838	1.00000	0.01303	-9284.8209		
	ND1	Model A	0.01382	0.96221	1.00000	0.03779	1.00000	0.00000	-7564.9954	0.0000	
		Null model	0.01382	0.96221	1.00000	0.03779	1.00000	0.00000	-7564.9954		
	ND2	Model A	0.04543	0.87657	1.00000	0.09880	10.21997	0.02214	-12295.1278	4.9813	150 I 0.964*, 234 L 0.969*
		Null model	0.04512	0.85531	1.00000	0.09589	1.00000	0.04388	-12297.6185		
	ND3	Model A	0.01993	0.86044	1.00000	0.13313	1.00000	0.00557	-3341.7873	0.0000	
		Null model	0.01983	0.85950	1.00000	0.13298	1.00000	0.00651	-3341.7873		
	ND4	Model A	0.03011	0.92879	1.00000	0.07121	1.00000	0.00000	-12672.0062	0.0000	377 V 0.967*
		Null model	0.03011	0.92879	1.00000	0.07121	1.00000	0.00000	-12672.0062		
	ND4L	Model A	0.02311	0.00958	1.00000	0.00120	1.00000	0.87881	-2748.8670	0.0000	
		Null model	0.02312	0.01430	1.00000	0.00180	1.00000	0.87410	-2748.8670		

	ND5	Model A	0.03779	0.89487	1.00000	0.09753	2.78643	0.00686	-17150.2974	0.4376	
		Null model	0.03779	0.89142	1.00000	0.09718	1.00000	0.01027	-17150.5162		
	ND6	Model A	0.02001	0.82946	1.00000	0.17054	1.00000	0.00000	-5535.5151	1.1533	
		Null model	0.01900	0.72270	1.00000	0.14919	1.00000	0.10619	-5534.9385		
Galatheoidea	ATP6	Model A	0.03484	0.00042	1.00000	0.00004	1.00000	0.92130	-3831.9678	0.0000	
		Null model	0.03484	0.00001	1.00000	0.00000	1.00000	0.92171	-3831.9678		
	ATP8	Model A	0.06121	0.62179	1.00000	0.37821	1.00000	0.00000	-1055.7877	0.0000	
		Null model	0.06121	0.62179	1.00000	0.37821	1.00000	0.00000	-1055.7877		
	COX1	Model A	0.00955	0.97839	1.00000	0.01392	1.00000	0.00758	-6627.4862	0.0000	
		Null model	0.00955	0.97839	1.00000	0.01392	1.00000	0.00758	-6627.4862		
	COX2	Model A	0.02356	0.93057	1.00000	0.03646	999.00000	0.03172	-3467.1838	2.0274	
		Null model	0.02377	0.91505	1.00000	0.03709	1.00000	0.04599	-3468.1975		
	COX3	Model A	0.02098	0.00000	1.00000	0.00000	1.00000	0.93574	-3990.1575	0.0000	
		Null model	0.02098	0.00013	1.00000	0.00001	1.00000	0.93560	-3990.1575		
	CYTB	Model A	0.02601	0.73893	1.00000	0.03483	999.00000	0.21606	-6026.4202	0.1193	
		Null model	0.02620	0.72678	1.00000	0.03448	1.00000	0.22793	-6026.4798		
	ND1	Model A	0.01805	0.93159	1.00000	0.03028	5.04976	0.03693	-4838.4537	4.0716	250 L 0.952*
		Null model	0.01783	0.92178	1.00000	0.03018	1.00000	0.04652	-4840.4896		
	ND2	Model A	0.03865	0.81617	1.00000	0.16570	6.28627	0.01507	-6757.0933	0.4062	168 S 0.956*
		Null model	0.03836	0.79317	1.00000	0.16243	1.00000	0.03685	-6757.2964		
	ND3	Model A	0.02249	0.85728	1.00000	0.14272	1.00000	0.00000	-1950.9258	0.0000	
		Null model	0.02249	0.85728	1.00000	0.14272	1.00000	0.00000	-1950.9258		
	ND4	Model A	0.02438	0.91810	1.00000	0.07386	0.00744	0.27986	-7191.2547	1.3189	
		Null model	0.02462	0.91651	1.00000	0.07480	1.00000	0.00803	-7191.9141		
	ND4L	Model A	0.01950	0.82253	1.00000	0.12081	999.00000	0.04940	-1642.7528	0.6995	
		Null model	0.01916	0.82126	1.00000	0.11478	1.00000	0.05612	-1643.1025		

ND5	Model A	0.03082	0.83473	1.00000	0.14950	54.93988	0.01337	-9934.4041	5.9443	88 F 0.979*, 473 S 0.960*
	Null model	0.03027	0.82024	1.00000	0.14580	1.00000	0.02883	-9937.3763		
ND6	Model A	0.04065	0.73557	1.00000	0.22230	748.37313	0.03235	-3631.2141	3.4159	
	Null model	0.04063	0.73510	1.00000	0.26490	1.00000	0.00000	-3632.9221		

* 0.95 < BEB < 0.99, ** BEB < 0.99.
